# Transcriptome Analysis Reveals the Immunoregulatory Effect of Two Polysaccharides from *Rhodomyrtus tomentosa*

**DOI:** 10.3390/foods15020235

**Published:** 2026-01-09

**Authors:** Dingjin Li, Qiuxia Duan, Wan Zunairah Wan Ibadullah, Radhiah Shukri, Hui Nie, Aiqing Ren, Nor Afizah Mustapha

**Affiliations:** 1 Guangxi Key Laboratory of Health Care Food Science and Technology, School of Food and Biological Engineering, Hezhou University, Hezhou 542800, China; lidingjin@hzxy.edu.cn (D.L.); qiuxiajiayou@163.com (Q.D.);; 2 Department of Food Technology, Faculty of Food Science and Technology, University Putra Malaysia (UPM), Serdang Selangor 43400, Malaysia; wanzunairah@upm.edu.my (W.Z.W.I.);

**Keywords:** *Rhodomyrtus tomentosa*, polysaccharides, immunomodulatory activity, transcriptome sequencing

## Abstract

The *Rhodomyrtus tomentosa* (Aiton.) Hassk. berry is rich in structurally diverse polysaccharides with potential biological activity. However, its immunomodulatory properties remain understudied, limiting our current understanding of its functional significance. Two structurally distinct polysaccharides from *Rhodomyrtus tomentosa* (RTP-1 and RTP-2) were evaluated for immunostimulatory activity in RAW264.7 macrophages. Phagocytic function was assessed by neutral red assay, nitric oxide (NO) and reactive oxygen species were measured using the Griess assay and fluorescent probes, and cytokines (TNF-α, IL-6 and IL-1β) were quantified by enzyme-linked immunosorbent assay. Analysis of RNA-seq data using weighted gene co-expression network analysis revealed co-expression modules. The selected transcripts were independently validated by quantitative real-time PCR (RT-qPCR). The results showed that both polysaccharides enhanced phagocytosis, increased NO/ROS levels, and promoted cytokine secretion. Transcriptome results indicated that RTP-2 activated the MEturquoise co-expression module containing 222 hub genes, whereas RTP-1 was mainly associated with the MECyan module containing 49 hub genes. Module enrichment for RTP-2 revealed links with mitophagy–immune regulation, proteostasis/stress, and innate immune signaling. RT-qPCR further confirmed that in the RTP-2 group, Dram1 expression was upregulated approximately 121 times, Bmf1 expression was upregulated approximately 18 times, and Bnip3 was significantly downregulated, whereas Bnip3l expression remained unchanged. Overall, RTP-2 exhibited a more pronounced and coherent macrophage-stimulating profile in vitro, supporting its potential as a macrophage-targeted immunostimulatory ingredient.

## 1. Introduction

The innate immune system is essential for host defense, with macrophages acting as key effector cells in immune surveillance and inflammatory regulation, typically accompanied by a metabolic shift toward enhanced glycolysis during activation [1]. Natural polysaccharides exhibit diverse biological functions, including antioxidant [2], anti-inflammatory [3], anticancer [4], and immunomodulatory activities [5]. Polysaccharides can activate macrophages by enhancing phagocytosis, promoting nitric oxide (NO) and reactive oxygen species (ROS) production, and stimulating cytokine secretion (such as TNF-α, IL-6, and IL-1β) [6]. Research indicates that the activating effects of polysaccharides on macrophages are closely related to their structural characteristics, including molecular weight, monosaccharide composition, glycosidic bond type, and higher-order conformation. A previous study reported that low-molecular-weight *Dendrobium officinale* polysaccharides induce stronger RAW264.7 macrophage activation than the high-molecular-weight polysaccharide [7]. Another study reported that an α-(1 → 4)-glucan-type polysaccharide from *Gastrodia elata* activated RAW264.7 macrophages by enhancing phagocytosis and upregulating inducible nitric oxide synthase (iNOS) and pro-inflammatory cytokines (TNF-α and IL-1β) [8]. In addition, triple-helical β-D-glucan from *Ganoderma lucidum* exhibits molecular-weight–dependent immunostimulatory activity, with higher-molecular-weight fractions inducing stronger cytokine release (IL-6 and TNF-α) in immune cell models [9]. However, systematic comparative studies of different polysaccharide fractions from the same plant source remain relatively scarce, limiting mechanistic insight into the immunomodulatory effects of structurally diverse polysaccharides on macrophages.

*Rhodomyrtus tomentosa* (Aiton.) Hassk. is a traditional medicinal and edible plant rich in bioactive polysaccharides [10]. In our previous work, we successfully isolated and structurally characterized two polysaccharides from *Rhodomyrtus tomentosa* fruit (RTP-1 and RTP-2). RTP-1 is a low molecular weight heteropolysaccharide (11.638 kDa) composed of mannose (55.43%), galactose (38.77%), and glucose (5.80%). RTP-1 composed of 4,6)-β-D-Manp-(1 → and 6)-β-D-Manp-(1 → linkages, and the branched chain is the D-Galp-(1 → attached to the O-6 position of the glycan residue → 4,6)-β-D-Manp-(1→) [11]. In contrast, RTP-2 has a higher molecular weight (29.058 kDa) and is composed mainly of glucose (74.29 L%), arabinose (10.54%), galactose (9.80%), and mannose (5.37%). It has a triple-helical conformation and a porous morphology. The RTP-2 backbone consists of (1 → 6)-α-D-Glcp, (1 → 4)-α-D-Glcp, (1 → 4,6)-α-D-Glcp, and (1 → 3,6)-β-D-Galp residues. Branching occurs primarily at the O-6 position (containing Araf and Glcp side chains) [12]. These significant structural differences provide a rational basis for direct comparison between RTP-1 and RTP-2. However, their differential immunoregulatory effects in macrophages and underlying molecular mechanisms remain poorly understood.

In this study, RAW264.7 cells (a murine macrophage-like cell line) were used to comparatively evaluate the immunomodulatory activities of RTP-1 and RTP-2 by assessing changes in phagocytosis, NO and ROS production, and cytokine secretion. We hypothesized that the pronounced structural divergence between RTP-1 and RTP-2 would result in distinct macrophage response profiles and different transcriptomic signatures. Transcriptome sequencing was further combined with weighted gene co-expression network analysis (WGCNA), protein–protein interaction (PPI) analysis, and RT–qPCR validation to identify candidate pathways and key genes associated with the differential responses. Overall, this study aimed to link structural features of RTP-1 and RTP-2 with their immunomodulatory potential, thereby providing a rationale for structure–function-guided development of natural immune modulators.

## 2. Materials and Methods

### 2.1. Materials and Reagents

Polysaccharides from *R. tomentosa* fruits were prepared following our previously reported procedure with minor modifications [11]. In brief, ripe fruits were purchased from a local market (Hezhou, Guangxi, China). Ultrasonic-assisted enzymatic extraction was used to obtain crude polysaccharides, followed by deproteinization, decolorization, ethanol precipitation, and lyophilization. The crude extract was fractionated using a DEAE-52 cellulose column. The water-eluted fraction was collected and purified on a Sephadex G-75 column to yield RTP-1. The 0.1 M NaCl-eluted fraction from the DEAE-52 column was purified on Sephadex G-75 to afford RTP-2. The total carbohydrate content of RTP-1 and RTP-2 was 86.03 ± 0.31% and 87.07 ± 0.91%, respectively. Protein and nucleic acids were detected in both samples.

RAW264.7 cells (murine macrophage-like cell line) were purchased from Nanjing Cobioer Biosciences Co., Ltd. (Nanjing, China) (Cat. No. CBP60533). Hifair^®^ AdanceFast One-step RT-gDNA Digestion SuperMix for qPCR, 2000 DNA Marker, and safe Green DNA Stain were purchased from Yeasen Biotechnology Co., Ltd. (Shanghai, China). Lipopolysaccharide (LPS) from *Escherichia coli O111:B4* was purchased from Beyotime Biotechnology Co., Ltd. (Shanghai, China). All other chemicals were purchased from Solarbio Science & Technology Co., Ltd. (Beijing, China).

### 2.2. Cell Culture

RAW264.7 macrophages were propagated in DEMM (pre-supplemented with penicillin 100 U/mL and streptomycin 100 μg/mL) containing 10% fetal bovine serum (FBS) at 37 °C in a humidified atmosphere with 5% CO_2_ atmosphere incubator (Thermofisher Scientific Inc., Allentown, PA, USA). RAW264.7 macrophages were seeded into 96-well or 6-well plates and cultured for 24 h to allow adherence. Subsequently, the medium was replaced with fresh DEME containing different concentrations of RTP-1 or RTP-2 (10, 40, and 160 μg/mL, respectively). Lipopolysaccharide (LPS, 10 μg/mL) was included as positive control [13], whereas untreated cells cultured under identical conditions served as the negative control [14]. The cells were incubated under the same conditions for the designated time periods until analysis. The polysaccharide concentrations were selected to span a commonly used working range in macrophage stimulation assays and to provide a low–mid–high gradient with 4 times spacing (10, 40 and 160 μg/mL) for dose–response evaluation.

### 2.3. Cell Viability Assay

The cell viability was determined by cell-counting kit-8 (CCK-8) assay [15]. RAW264.7 macrophages were seeded into 96-well plates at a density of 1 × 10^5^ cells/mL (100 μL/well) and incubated for 24 h to allow adherence. The medium was then replaced with fresh DEME containing RTP-1 or RTP-2 (10, 40, and 160 μg/mL). Lipopolysaccharide (LPS, 10 μg/mL) was used as the positive control, and untreated cells were used as the negative control. After 24 h of treatment, 10 μL of the CCK-8 solution was added to each well and incubated for 1 h. The absorbance was measured at 450 nm using a microplate reader. The cell viability and cell inhibition rate were calculated as follows:

(1) $$\mathrm{Cell}\;\mathrm{viability}\;\left(\%\right)\;=\frac{A_{1}}{A_{2}}\times 100\%$$

where A_1_ is the absorbance of the sample group and A_2_ is the absorbance of the control group.

### 2.4. Phagocytic Activity of Cells

The phagocytic capacity of macrophages was measured by neutral red assay [16]. RAW264.7 macrophages were seeded into 96-well plates at 1 × 10^5^ cells/mL and treated with RTP-1 or RTP-2 at various concentrations (10, 40, and160 μg/mL). Lipopolysaccharide (LPS, 10 μg/mL) was used as the positive control, and untreated cells were used as the negative control. After 24 h of treatment, 100 μL of 0.01% neutral red solution was added to each well and incubated for 2 h. The supernatant was then removed, and the cells were washed with PBS and lysed with cell lysis buffer to eliminate excess dye. After shaking for 10 min at room temperature, the absorbance was measured at 540 nm using a microplate reader.

### 2.5. Determination of Nitric Oxide (NO)

RAW264.7 cells (1 × 10^5^ cells/mL, 200 μL/well) were added to 96-well plates and treated with RTP-1 or RTP-2 at concentrations of 10, 40, and 160 μg/mL. Untreated cells were used as the negative control, and LPS (10 μg/mL) as the positive control. The nitric oxide (NO) levels in the culture supernatants were determined after 24 h of incubation using a commercial NO assay kit according to the manufacturer’s instructions.

### 2.6. Measurement of Reactive Oxygen Species (ROS)

Intracellular reactive oxygen species (ROS) levels were assessed using the fluorescent probe DCFH-DA. RAW264.7 cells (2 × 10^5^ cells/mL, 1 mL/well) were seeded in 12-well plates and treated with RTP-1 and RTP-2 (10, 40, and 160 μg/mL) or LPS (10 μg/mL) for 24 h. Subsequently, cells were incubated with DCFH-DA (10 μM) for 30 min at 37 °C in the dark, washed with PBS, and analyzed by flow cytometry to measure mean fluorescence intensity (MFI) according to the manufacturer’s instructions.

### 2.7. Determination of Cytokine

The RAW264.7 macrophages (1 × 10^5^ cells/mL) were cultured in a 48-well plate. Cells were then treated with RTP-1 and RTP-2 (10, 40, and 160 μg/mL) or LPS (10 μg/mL, positive control) for 24 h at 37 °C. The concentrations of TNF-α, IL-1β, and IL-6 in the culture supernatants were determined using commercial enzyme-linked immunosorbent assay kits (Beijing Solarbio Science & Technology Co., Ltd., Beijing, China): TNF-α ELISA Kit (SEKM-0034; lower limit of detection: 3.9 pg/mL), IL-6 ELISA Kit (SEKM-0007; lower limit of detection: 3.9 pg/mL), and IL-1β ELISA Kit (SEKM-0002; lower limit of detection: 15.63 pg/mL), according to the manufacturer’s instructions.

### 2.8. Total RNA Extraction

RAW264.7 macrophages were seeded in 6-well plates at a density of 2 × 10^6^ cells/mL and treated with 40 μg/mL RTP-1 (group B) or RTP-2 (group C) for 24 h at 37 °C in a humidified 5% CO_2_ incubator. The concentration (40 μg/mL) was selected based on functional screening because it elicited robust macrophage activation and showed clear differences between RTP-1 and RTP-2 across key readouts (NO/ROS production and cytokine secretion), while remaining within a non-cytotoxic window. The 24 h was chosen to align with the endpoint design used in the in vitro assays and capture an integrated transcriptional response associated with sustained activation. Cells cultured in complete medium without treatment were used as the negative control (group A). Each group contained three independent biological replicates (A1–A3, B1–B3, and C1–C3). These biological replicates were derived from independent cell culture systems and processed separately. During RNA extraction and subsequent library preparation, sample processing order was randomized to minimize technical variation. After incubation, the medium was discarded, and the cells were washed twice with diethyl pyrocarbonate (DEPC)-treated PBS. Total RNA was extracted by adding 1 mL of TRIzol reagent to each well, followed by thorough pipetting to ensure complete cell lysis and homogenization.

The lysates were transferred to RNase-free 1.5 mL tubes, and chloroform was added. After vigorous shaking, the samples were incubated at room temperature for 3 min and centrifuged at 12,000 rpm for 15 min at 4 °C. The upper aqueous phase was carefully collected and transferred to a new RNase-free tube, and an equal volume of isopropanol was added to precipitate the RNA. After incubation at room temperature for 10 min, the mixture was centrifuged at 12,000 rpm for 15 min at 4 °C. The RNA pellet was washed with 75% ethanol, centrifuged at 12,000 rpm for 15 min at 4 °C, and air-dried. Finally, the pellet was dissolved in water treated with DEPC for subsequent analysis [17].

### 2.9. mRNA Purification, Library Construction and Sequencing

Total RNA was purified from each sample using Oligo(dT) magnetic beads to enrich polyadenylated mRNA. The isolated mRNA was fragmented by ion-mediated methods and used as a template for complementary DNA (cDNA) synthesis. The resulting cDNA was amplified by PCR to construct sequencing libraries. Library quality was assessed by Agilent 2100 Bioanalyzer (Agilent Technologies, Inc., Santa Clara, CA, USA), and concentrations were quantified by fluorescence-based assays. Finally, libraries at the optimal concentration were subjected to paired-end sequencing on an Illumina HiSeq X10 platform. The experiment included three biological replicates per group. To minimize technical bias, all samples were randomized during library preparation and sequenced on the same flow cell using the Illumina HiSeq X10 platform to avoid batch effects [17].

### 2.10. Weighted Gene Co-Expression Network (WGCNA) Analysis

Weighted Gene Co-expression Network Analysis (WGCNA) was performed to construct unsigned co-expression networks and identify modules of co-expressed genes. Module eigengenes (MEs) were correlated with phenotypic traits using Pearson’s correlation to determine trait-associated modules. Genes from the most relevant modules were selected for the downstream analysis. Functional enrichment of candidate genes was conducted using the Search Tool for the Retrieval of Interacting Genes/Proteins (STRING, version 2) database, and a protein–protein interaction (PPI) network was generated. Clusters within the PPI network were identified using the Markov Cluster Algorithm (MCL), and the top three clusters with the highest connectivity and functional relevance were subjected to pathway enrichment analysis to reveal key biological processes [18].

### 2.11. Real-Time Quantitative Polymerase Chain Reaction (RT-qPCR) Validation

Total RNA was extracted and reverse-transcribed into complementary DNA (cDNA) using a commercial first-strand cDNA synthesis kit. RT-qPCR was performed using SYBR Green chemistry, with each sample analyzed in technical triplicate. Glyceraldehyde-3-phosphate dehydrogenase (GAPDH) was used as the internal reference gene, and its expression stability across control and treatment groups was verified prior to normalization. The cycling conditions were as follows: initial denaturation at 95 °C for 2 min, followed by 40 cycles of denaturation at 95 °C for 10 s and annealing/extension at 60 °C for 30 s. A melting curve analysis was performed from 60 °C to 95 °C at a ramp rate of 0.05 °C/s. The primer sequences are listed in Table 1. Relative gene expression levels were calculated using the 2^−ΔΔC^^T^ method [19].

### 2.12. Statistical Analysis

All experiments were performed using three independent biological replicates (*n* = 3). Data are presented as mean ± SD. Differences among groups were analyzed by one-way ANOVA, followed by Duncan’s multiple range test for post hoc comparisons. A value of *p* < 0.05 was considered statistically significant.

## 3. Results and Discussion

### 3.1. Cell Viability 

**Figure 1:**
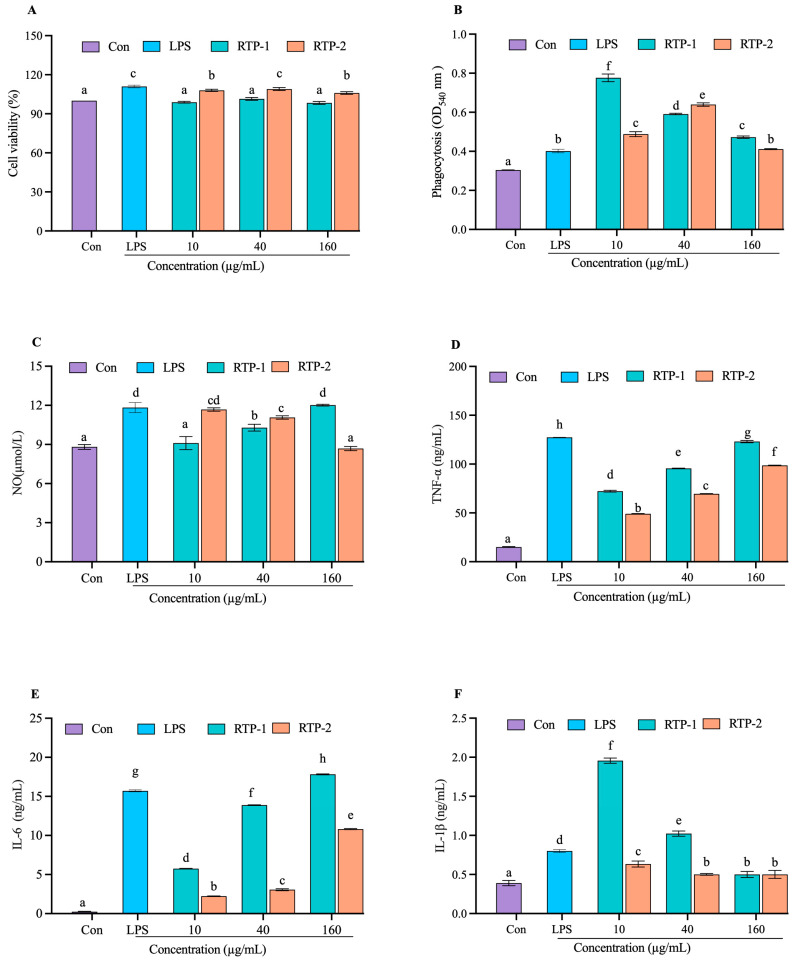
Immunomodulatory activity of RTP-1 and RTP-2 on RAW264.7. (**A**) cells viability, (**B**) phagocytic activity, (**C**) NO production, (**D**), TNF-α (**E**) IL-6, (**F**) IL-1β. Different letters indicated a different significance among all the groups at *p* < 0.05.

The CCK-8 assay is widely used to assess cell viability and cytotoxicity [20]. As shown in Figure 1A, both RTP-1 and RTP-2 exhibited excellent biocompatibility within the concentration range of 10–160 μg/mL. Cell viability in all treated groups remained between 95% and 110%, with no significant cytotoxic effects compared with the control group. Importantly, this effect was concentration-independent, as viability remained high even at the maximum tested dose. These results demonstrate that RTP-1 and RTP-2 are non-cytotoxic and have good safety characteristics, providing a reliable basis for subsequent biological function studies.

### 3.2. Phagocytic Activity

Phagocytosis is a critical component of the innate immune defense, primarily mediated by macrophages and neutrophils to eliminate pathogens and cellular debris [21]. Therefore, enhanced phagocytic activity is a hallmark of immune stimulation and an important indicator of immunoenhancing potential [22]. As shown in Figure 1B, both RTP-1 and RTP-2 significantly enhanced the phagocytic activity of RAW264.7 macrophages. RTP-1 exerted the strongest effect at 10 μg/mL (OD_540_ = 0.78), which was significantly higher than that observed in all other treatment groups. At 40 μg/mL, RTP-2 also significantly increased phagocytosis (0.63 ± 0.01) compared with both unstimulated and LPS-treated controls (*p* < 0.01). This indicates that moderate doses of RTP-2 effectively activate macrophage phagocytosis, possibly by stimulating cell-surface receptors or enhancing intracellular signaling pathways [23]. Phagocytic activity decreased at higher concentrations (0.41 ± 0.01 at 160 μg/mL) but remained significantly higher than that of the LPS control group (*p* < 0.05). One of the defining features of activated macrophages is their enhanced phagocytic [24]. Collectively, these findings indicate that RTP-1 and RTP-2 strengthen innate immune defenses by boosting macrophage phagocytosis, thereby supporting their potential as natural immune enhancer.

### 3.3. Determination of Nitric Oxide Release

Nitric oxide (NO) is mainly produced by inducible nitric oxide synthase (iNOS) in activated macrophages. It is an important effector molecule that promotes pathogen clearance, tumor suppression, and macrophage lytic and phagocytic functions [25]. Thus, NO production is a reliable indicator of macrophage activation and immunoenhancement [26]. Figure 1C shows that RTP-1 significantly stimulated NO release in a concentration-dependent manner. Compared with the control, NO production was not significantly altered at 10 μg/mL. However, it significantly increased at 40 and 160 μg/mL, indicating macrophage activation.

Interestingly, RTP-2 exhibited a stronger potency at lower doses. At 10 μg/mL, RTP-2 induced NO levels comparable to those achieved by 160 μg/mL of RTP-1, indicating higher immunostimulatory efficiency. However, NO production gradually declined at higher RTP-2 concentrations, and at 160 μg/mL, it was not significantly different from the control at 160 g/mL. This trend implies that RTP-2 may activate NO synthesis through distinct mechanisms, potentially involving receptor saturation, polysaccharide aggregation, or negative feedback regulation to avoid excessive immune stimulation [27]. Although both RTP-1 and RTP-2 promote nitric oxide production, their different dose–response patterns indicate that they exert regulatory effects through different immunomodulatory mechanisms.

### 3.4. Determination of Reactive Oxygen Species

**Figure 2:**
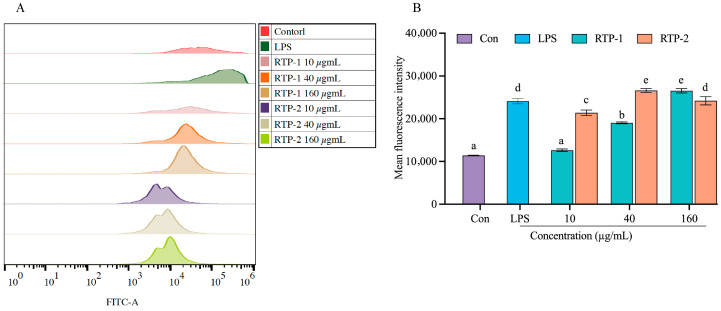
Effects of RTP-1 and RTP-2 on ROS generation (**A**,**B**). Different letters indicated a different significance among all the groups at *p* < 0.05.

Reactive oxygen species (ROS) are key markers of macrophage activation. Upon stimulation, macrophages rapidly generate reactive oxygen species (ROS), which not only contribute to direct pathogen clearance but also activate downstream immune signaling pathways [28]. Intracellular reactive oxygen species (ROS) levels are important indicators of immunostimulatory activity [29]. RTP-1 exerted a concentration-dependent effect on the production of ROS in RAW264.7 cells (Figure 2). At 10 μg/mL, ROS levels did not differ significantly from the control (*p* > 0.05). At 40 and 160 μg/mL, ROS generation increased significantly. This indicated that it may exert an immune-activating effect by regulating macrophages’ oxidative stress response (Sun et al., 2024, [30]). In contrast, RTP-2 displayed a different trend. It significantly elevated ROS production at both 10 and 40 μg/mL (*p* < 0.01), demonstrating a stronger stimulatory effect at lower doses. Although ROS levels declined at 160 μg/mL compared to 40 μg/mL, they remained significantly higher than the control (*p* < 0.05) and were comparable to those induced by LPS, suggesting that RTP-2 may trigger negative feedback regulation that limits ROS accumulation and maintains cellular redox balance. Notably, at equivalent or lower doses, RTP-2 induced higher ROS levels than RTP-1, and at 40 μg/mL, its effect matched or even exceeded that of 160 μg/mL RTP-1. Overall, RTP-2 induced higher ROS levels than RTP-1 at comparable or lower concentrations, indicating a more pronounced ROS response in RAW264.7 macrophages.

### 3.5. Determination of Tumour Necrosis Factor (TNF-α) Levels

Tumor necrosis factor-α (TNF-α) is a central cytokine produced by activated macrophages that strengthens host defense by stimulating immune cell activation, promoting their recruitment to sites of infection, and supporting pathogen clearance. In addition to its roles in inflammation, tumor suppression, and metabolic regulation, elevated TNF-α production is a key marker of macrophage activation and immunoenhancing activity [31]. As shown in Figure 1D, both RTP-1 and RTP-2 significantly increased TNF-α secretion compared with the control across all tested concentrations, showing a concentration-dependent pattern. At 160 μg/mL, RTP-1 induced TNF-α levels that were comparable to those of the LPS-treated group under our experimental conditions. RTP-2 also elevated TNF-α secretion, although the magnitude of the increase was lower than that observed for RTP-1 at the corresponding doses. Overall, RTP-1 and RTP-2 elicit TNF-α–associated activation responses in RAW264.7 macrophages

### 3.6. Determination of Interleukin-6 (IL-6) Levels

Interleukin-6 (IL-6) is a multifunctional cytokine that plays a central role in immune regulation. It participates in the acute-phase response, promotes B- and T-cell differentiation, supports antibody production, and contributes to tissue repair and host defense. As shown in Figure 1F, both RTP-1 and RTP-2 significantly increased IL-6 secretion in RAW264.7 macrophages compared with that in the control. RTP-1 showed a concentration-dependent increase, with IL-6 levels rising from 10 μg/mL to 40 μg/mL and approaching the LPS-treated group at 40 μg/mL under our experimental conditions. RTP-2 also elevated IL-6 production across all tested concentrations, with a relatively small change in magnitude and a relatively stable response pattern. Overall, RTP-1 and RTP-2 elicit IL-6–associated activation responses in RAW264.7 macrophages, with RTP-1 producing a stronger IL-6 response than RTP-2 at comparable doses.

### 3.7. Determination of Interleukin-1β (IL-1β) Levels

Interleukin-1β (IL-1β) is a proinflammatory cytokine mainly produced by activated macrophages. IL-1β serves as a key mediator in initiating and amplifying immune responses, recruiting effector cells to infection sites, and enhancing pathogen clearance [32]. IL-1β secretion is an important marker of macrophage activation and immune enhancement [33]. At 10 μg/mL, RTP-1 significantly increased IL-1β secretion (Figure 1F). In contrast, IL-1β levels at 40 and 160 μg/mL were reduced compared with 10 μg/mL, indicating a concentration-dependent modulation of IL-1β production by RTP-1 in RAW264.7 macrophages. In contrast, RTP-2 induced a relatively stable increase in IL-1β secretion across the tested concentrations, with a smaller magnitude variation than RTP-1. This differential response pattern likely arises from structural variations or distinct modes of action between the two polysaccharides, suggesting that RTP-1 and RTP-2 may regulate IL-1β production through different cellular signaling pathways.

### 3.8. Results of RNA Quality Testing

According to Table 2, the RNA concentration, total amount and completeness of all the samples met the requirements for library construction and sequencing, and the assessment grades were all A, indicating that the samples were of good quality and could be used for subsequent library construction and sequencing analysis.

### 3.9. Sample Clustering and Outliers’ Detection

**Figure 3:**
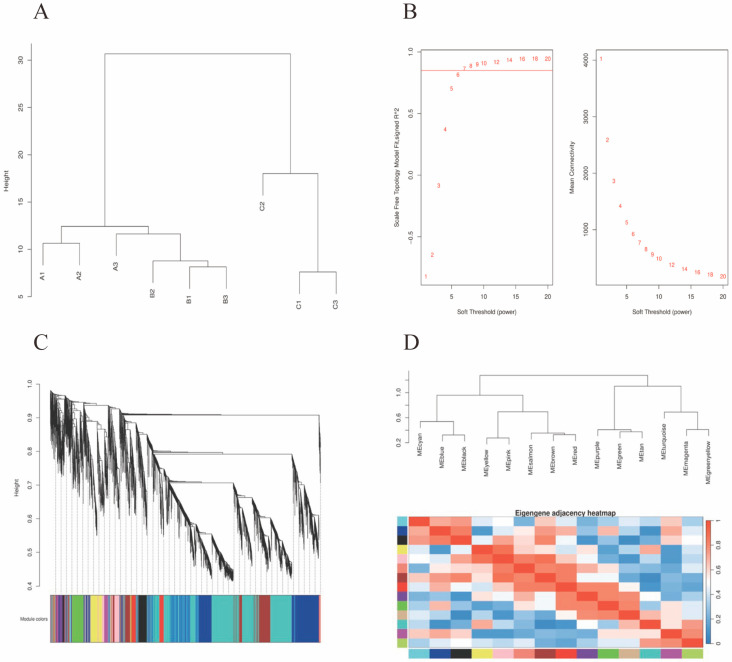
Sample clustering (**A**), soft-threshold selection (**B**), module construction (**C**), and eigengene correlations (**D**).

Hierarchical clustering analysis was performed using gene expression profiling data. The Euclidean distance was used to measure the differences between the samples, and the clustering dendrogram was constructed using the Average Linkage method (Figure 3A). The results showed that the nine samples (A1–A3 for control group, B1–B3 for RTP-1 group, and C1–C3 for RTP-2 group) were stably clustered in their respective experimental groups, and no obvious outlier samples were observed (the heights of the branches in the dendrograms did not exceed the preset thresholds), indicating that the batch effect between experiments was low. The results showed that the batch effect between experiments was small, and the data had good consistency and reproducibility, which could meet the requirements of further research.

### 3.10. Soft Threshold Screening

The power-law distribution properties of network connectivity for different soft thresholds (β) were evaluated using a scale-free topology fit index (β). The minimum value of β is chosen such that the fit index was greater than 0.85, while ensuring that the mean connectivity did not significantly decrease. As shown in Figure 3B, the fitting index reached 0.89 (R^2^ = 0.85) for the soft threshold β = 7, which meets the requirement of scale-free networks, and the distribution of intergenic linkage weights under this parameter is in line with the biological co-expression characteristics. In this analysis, after checking the grouping information and the presence of abnormal samples by plotting the sample clustering, a soft threshold value of 7 was obtained, which meets the value of the scale-free network and can be continued for subsequent analyses.

### 3.11. WGCNA Analysis

#### 3.11.1. Gene Clustering and Dendrogram Construction

Figure 3C illustrates the co-expression network constructed using the WGCNA method. All genes in the expression profile were filtered, and the top 65% of genes based on the median absolute deviation (MAD) were selected, resulting in 9827 differentially expressed genes. These genes were further clustered into 52 co-expression modules, with the gray module representing genes that could not be assigned to any specific module. This result indicates significant gene co-expression patterns within the dataset, suggesting that different modules play roles in specific biological functions or regulatory pathways. Subsequent analysis can involve correlating these modules with phenotypic traits to identify key modules significantly associated with specific traits, followed by the identification of key genes within these modules and functional enrichment analysis, thereby revealing potential regulatory mechanisms and biological significance involved in the response to immune stimulants.

#### 3.11.2. Heatmap of Module Eigengene Correlations

Based on the similarity of gene expression levels, a weighted gene co-expression network was constructed using the WGCNA method. Genes with similar expression patterns were grouped into modules, and similar modules were merged to enhance network stability. The resulting module–module correlation heatmap reveals a systematic co-expression structure of the transcriptome under polysaccharide stimulation (Figure 3D). Modules, distinguished by different colour codes, represent relatively independent or functionally coordinated gene clusters. The clear correlation gradient among modules suggests that polysaccharide-induced immune stimulation may activate multiple gene modules with distinct functional roles, laying the foundation for subsequent identification of key functional modules, screening of core regulatory genes, and elucidation of immune regulatory mechanisms.

#### 3.11.3. Module-Trait Association Analysis

**Figure 4:**
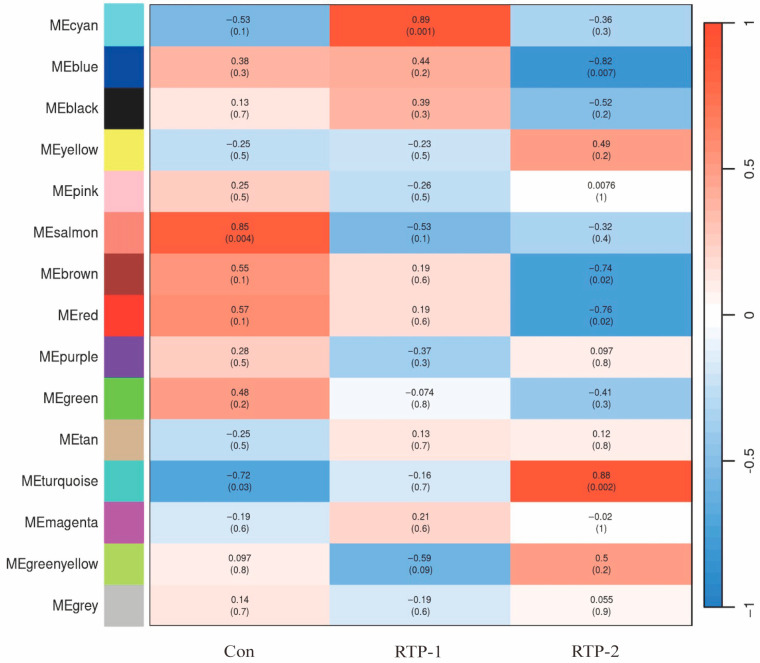
Modules and phenotype association.

To systematically reveal the association between gene expression and phenotypic changes under polysaccharide intervention, weighted gene co-expression network analysis (WGCNA) was used to construct module-trait association maps (Figure 4). The results revealed relatively weak correlations between the RTP-1 intervention group and the key modules. For example, the MEturquoise module exhibited only a slight negative correlation (R = −0.16). In contrast, in the RTP-2 treatment group, this module was highly positively correlated with trait (R = 0.88, *p* = 0.002), indicating that it may be an important response module under RTP-2 intervention. Furthermore, modules such as MEcyan (R = −0.53), MEblue (R = −0.57), and MEblack (R = −0.62) exhibited negative correlations with RTP-2 intervention, indicating that RTP-2 may be involved in the suppression of specific metabolic or stress-related pathways. In contrast to RTP-2, the MEcyan module exhibited a strong positive correlation with the RTP-1 intervention (R = 0.89, *p* = 0.001), indicating that RTP-1 activated this module.

This parallel pattern of activation and repression reflects the bidirectional regulatory effects of polysaccharide intervention on multiple functional networks at the transcriptional level. Comparative analyses of module-specific differences further support this observation, demonstrating that the two polysaccharides may differ in their mechanisms of receptor recognition and signalling activation because of structural differences, leading to different modes of action.

#### 3.11.4. Analysis of Module Membership and Gene Significance

**Figure 5:**
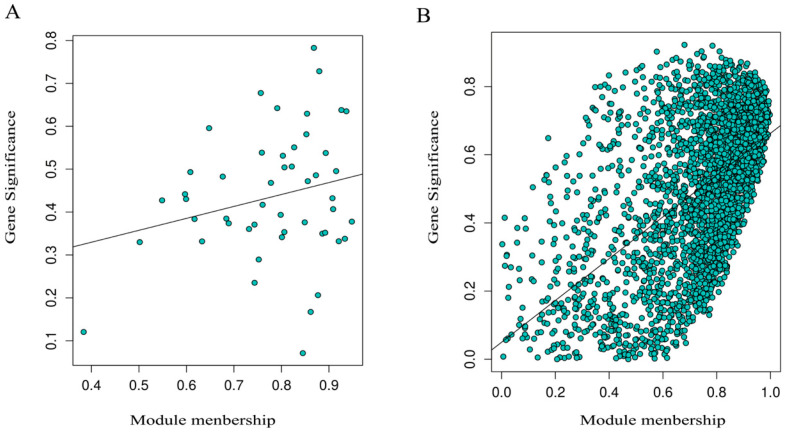
Modules and phenotype association ((**A**): RTP-1; (**B**): RTP-2).

During the identification of key regulatory genes, the MEturquoise module was selected as the representative module responsive to RTP-2 for further analysis of gene importance (Figure 5B). Using dual thresholds of gene significance (GS > 0.75) and module membership (MM > 0.75), 222 core candidate genes were identified. These genes occupy central topological positions within the module, and their expression levels show a high concordance with the treatment phenotype, indicating their potential roles as key drivers of immune regulation. In contrast, the MECyan-positive correlation module in the RTP-1 group screened only 49 core candidate genes (Figure 5A). The network connectivity and regulatory breadth of this group were significantly lower than those of the RTP-2 group, further demonstrating the superior immunomodulatory effect of RTP-2. Subsequently, these core genes were submitted to the STRING database to construct a protein–protein interaction network, revealing a high degree of connectivity. Several hub genes formed densely interconnected clusters, indicating potential functional synergy. Preliminary results indicate that genes within this module are highly enriched in immune recognition, transcriptional regulation, and metabolic pathways, providing valuable directions for future mechanistic studies.

#### 3.11.5. STRING Pathway Enrichment Analysis

**Figure 6:**
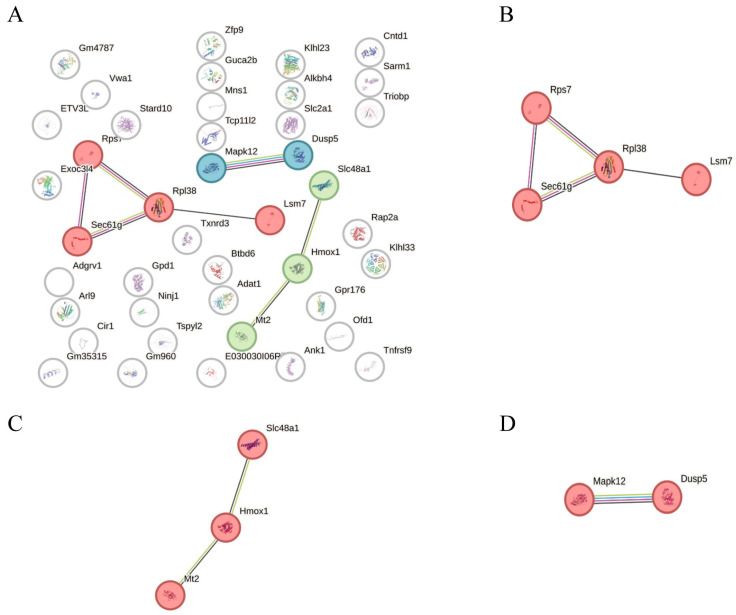
STRING pathway enrichment analysis of RTP-1. (**A**): Overall PPI network, (**B**): The top-ranked cluster, (**C**): The second-ranked cluster, (**D**): The third-ranked cluster.

**Figure 7:**
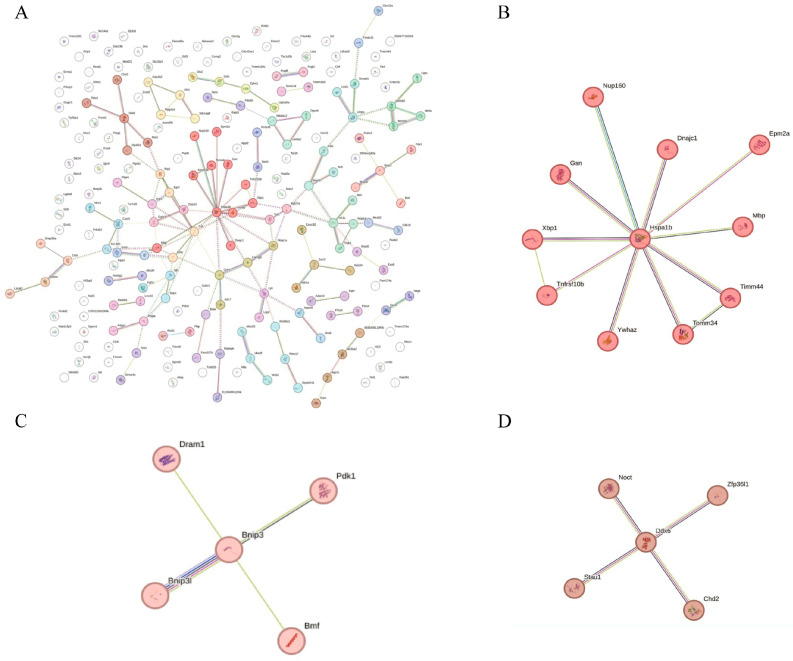
STRING pathway enrichment analysis of RTP-2. (**A**): Overall PPI network, (**B**): The top-ranked cluster, (**C**): The second-ranked cluster, (**D**): The third-ranked cluster.

Search tool for the retrieval of interacting genes/proteins (STRING) pathway enrichment analysis is a functional annotation method based on protein interaction networks that identifies the enrichment features of target genes or proteins in known biological pathways, thereby revealing the biological processes and regulatory networks in which they may be involved. To systematically investigate the biological functions of the above core genes, protein–protein interaction (PPI) analysis and pathway enrichment analysis were performed for 49 core genes in the RTP-1 significantly associated cyan module eigengene (MECyan) and 222 core genes in the RTP-2 significantly associated turquoise module eigengene (MEturquoise) using the STRING database. The Markov cluster algorithm (MCL) was employed to divide the RTP-1 and RTP-2 modules into 3 clusters (Figure 6) and 42 clusters (Figure 7), respectively.

As for RTP-2, the first-ranked cluster exhibited a star-like topology centered on heat shock protein A1b (Hspa1b), a classical molecular chaperone involved in protein folding, stabilization, and cellular stress responses. This central node was directly connected to multiple stress-associated proteins, including DnaJ heat shock protein family (Hsp40) member C1 (Dnajc1: a co-chaperone in the Hsp70 system), X-box binding protein 1 (Xbp1: a key transcription factor in the endoplasmic reticulum stress response), and translocase of outer mitochondrial membrane 34 (Tomm34) and translocase of inner mitochondrial membrane 44 (Timm44: mitochondrial translocases responsible for protein import into the outer and inner mitochondrial membranes, respectively). Additionally, proteins such as Nucleoporin 160 (Nup160: a nucleoporin mediating nucleocytoplasmic transport), Gelsolin (Gsn: an actin-binding protein regulating cytoskeletal remodeling), and tyrosine 3-monooxygenase/tryptophan 5-monooxygenase activation protein zeta (Ywhaz: a signaling adaptor protein involved in cell cycle control and apoptosis) were identified within the same cluster, suggesting integration of stress signaling, mitochondrial function, and cytoskeletal regulation. The presence of tumor necrosis factor receptor superfamily member 10b (Tnfrsf10b: a death receptor involved in apoptosis), myelin basic protein (Mbp: essential for neuronal function), and Epm2a (a glycogen metabolism regulator implicated in Lafora disease) further supports the hypothesis that this cluster represents a multifunctional stress-responsive module. Collectively, the molecular architecture of this firstly ranked network implies that RTP-2 may modulate cellular homeostasis under inflammatory or metabolic stress through coordinated regulation of protein quality control, mitochondrial dynamics, and intracellular signaling pathways.

The second-ranked cluster is centered on BCL2/adenovirus E1B 19 kDa-interacting protein 3 (Bnip3), which constitutes a tightly interconnected regulatory module encompassing key regulators of autophagy and apoptosis, such as DNA damage regulated autophagy modulator 1 (Dram1), Bcl2 modifying factor (Bmf1), BCL2/Adenovirus E1B 19 kDa-interacting protein 3-lik (Bnip3l), and pyruvate dehydrogenase kinase 1 (Pdk1) (Figure 7). This network may mediate the metabolic reprogramming of immune cells and drive the coordinated regulation of immune stimulation and functional activation. Specifically, Bnip3-driven and Bnip3l-driven mitochondrial autophagy promotes the clearance of damaged mitochondria and supports the metabolic shift toward glycolysis in immune cells, a metabolic transition that is a key feature of macrophage and dendritic cell activation. Pdk1 enhances glycolytic metabolic flow by inhibiting pyruvate dehydrogenase activity and directing glucose metabolism to branch toward the pentose phosphate pathway, enhancing NADPH production and providing energetic support for reactive oxygen species (ROS) production. This metabolic transition provides the necessary metabolic intermediates and energy base for cytokine synthesis and secretion. The moderate regulation of mitochondrial function and ROS production helps activate multiple signaling pathways and promote the expression of immune-related genes [34]. Bnip3-mediated mitochondrial remodeling plays an important role in regulating ROS levels, and the appropriate amount of ROS signals activates transcription factors and promotes the expression of immune factors. In addition, mitochondrial autophagy removes damaged mitochondria, preventing excessive ROS accumulation and maintaining the function and survival of immune cells. In addition, the synergistic action of Bmf1 with the Bnip3 axis promotes immunogenic cell death (ICD) and enhances antigen presentation and immunoreactivity through the release of damage-associated molecular patterns (DAMPs), such as ATP and HMGB1. Involvement of Dram1, on the other hand, indicates that autophagic processes augment MHC-II-mediated antigen presentation and promote the processing of exogenous and endogenous antigens, as well as T-cell activation.

The third-ranked cluster had Sarm1 as its core and formed a complex network containing multifunctional regulatory molecules such as Rnf4, Cnot2, and Slc39a1. As an important regulator of innate immune signaling, Sarm1 interacts with the ubiquitin ligase Rnf4 and the transcriptional regulatory complex component Cnot2, demonstrating that RTP-2 may finely regulate the expression of immune-related genes by modulating protein ubiquitination modifications and mRNA stability. This regulatory mechanism contributes to the enhancement of the activation state and functional response of immune cells, thereby promoting immune stimulation.

**Figure 8:**
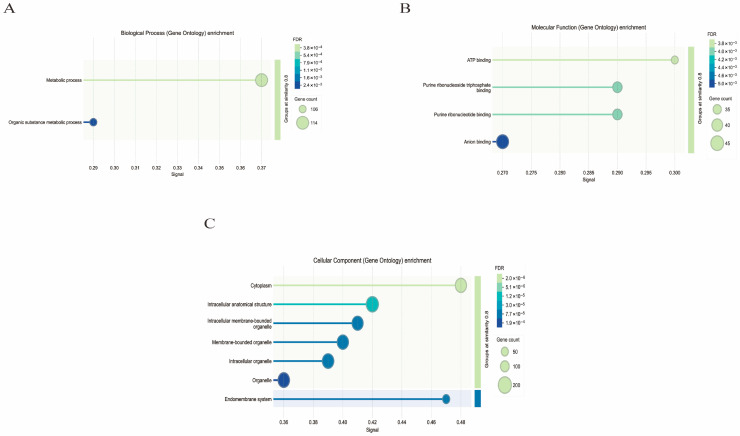
Gene Ontology (GO) enrichment analysis of differentially expressed genes in RTP-2 ((**A**): Biological process; (**B**): Molecular function; (**C**): Cellular component).

Biological process (Gene Ontology) enrichment analysis (Figure 8A) revealed significant enrichment in terms related to protein ubiquitination and proteolytic activity, indicating that RTP-2 may activate protein quality control and degradation mechanisms. These functions are essential for maintaining cellular homeostasis and eliminating damaged proteins [35]. Notably, the enrichment of membrane-associated ubiquitin ligase activity suggests a role for the polysaccharide in modulating the stability and signaling of membrane receptors, thereby influencing immune cell activation.

Molecular function (Gene Ontology) enrichment analysis (Figure 8B) revealed significant ATP binding, highlighting the central role of energy metabolism in polysaccharide 2-mediated immune regulation. The observed enrichment of protease inhibitor activity and serine-type endopeptidase activity indicated that RTP-2 helps maintain intracellular protein homeostasis by finely regulating the protein hydrolysis system. In addition, enhanced pyruvate dehydrogenase activity was directly correlated with glycolysis and the tricarboxylic acid (TCA) cycle, indicating that RTP-2 regulates immune cell metabolism to meet their functional requirements.

Cellular Component (Gene Ontology) enrichment analysis (Figure 8C) revealed a pronounced subcellular localization preference. Significant enrichment in intracellular vesicle membranes, membrane-bound organelles, and vesicular structures indicates that RTP-2 exerts its effects primarily through modulation of the endomembrane system and vesicular trafficking. Although the enrichment of neuronal projections and dendrites may initially seem unrelated to immune function, it likely reflects the structural remodeling requirements of immune cells (particularly dendritic cells and macrophages) during antigen presentation and morphological adaptation. Enrichment of endoplasmic reticulum membrane components further supports the involvement of RTP-2 in protein folding, post-translational modifications, and secretion processes.

In contrast, the PPI network structure of RTP-1 exhibited significantly different features. Its first-ranked family is centered on Rpl38, which connects proteins such as Rps7, Sec61g, and Lsm7, which are mainly involved in ribosome function and protein synthesis-related components. The relative simplicity of this network structure, which lacks the complex signaling regulatory hierarchy observed in RTP-2, indicates that RTP-1 acts mainly by regulating the underlying protein synthesis process rather than specific immune signaling pathway activation. In terms of functional integration, the gene network activated by RTP-1 exhibits low signaling connectivity. It is mainly associated with non-specific biological processes such as basal metabolism and cellular homeostasis. This structural feature is also consistent with the limited number of candidate genes identified in its response module (MECyan) and the lack of significant enrichment results in gene ontology (GO) and Kyoto Encyclopaedia of genes and genomes (KEGG), indicating a relatively limited immunostimulatory potential.

Comprehensive WGCNA modularity analysis, core gene screening with STRING protein interaction network and GO function enrichment results showed that the RTP-2 polysaccharide-treated group exhibited stronger immunoregulatory potential at the transcriptional level. The significantly activated MEturquoise module not only covered a large number of core genes, but also showed a high degree of network interconnectivity, focusing on several key pathways involved in stress sensing, protein quality control, mitochondrial function, autophagy and immune activation. Representative molecules such as Hspa1b, Xbp1, Bnip3, Bmf1, and Dram1 play central regulatory roles in the functional network constructed by RTP-2, constituting a multidimensional immune regulatory system centered on stress adaptation, metabolic reprogramming and antigen presentation. In contrast, the MECyan module activated by RTP-1 is relatively limited, the number of core genes is small, the network structure is biased towards the basic protein synthesis process, and it lacks a close connection with specific immune signaling pathways. These findings suggest that the two polysaccharides may exert distinct immunomodulatory profiles.

### 3.12. Real-Time Fluorescence Quantitative PCR Validation

**Figure 9:**
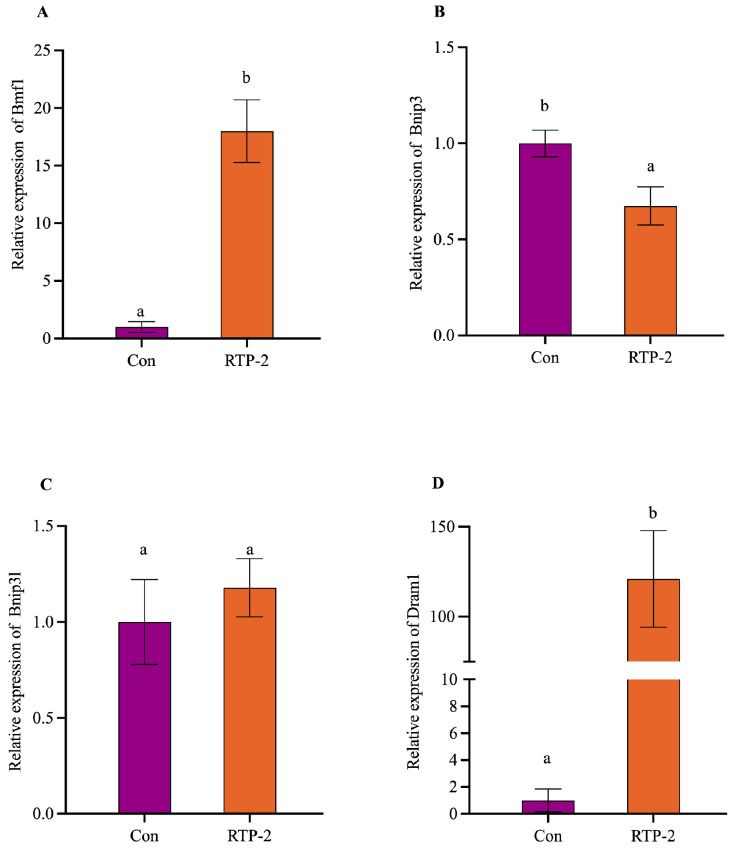
Effects of RTP-2 on the mRNA expression of autophagy/mitophagy-related genes ((**A**): Bmf1, (**B**): Bnip3, (**C**): Bnip3l, (**D**): Dram1). Different letters above the bars indicate significant differences among groups (*p* < 0.05).

To verify the reliability of the transcriptome analysis, representative genes (Dram1, Bmf1, Bnip3, and Bnip3l) closely associated with mitochondrial function, autophagy, and immune regulation were selected from the GO enrichment results of the RTP-2 samples for validation by RT-qPCR. The RT-qPCR results (Figure 9) indicated that.

Dram1 and Bmf1 mRNA expression levels were significantly upregulated compared with the control (*p* < 0.01), with 2^−ΔΔCT^ values of 120.99 ± 20.88 and 17.99 ± 2.71, respectively. The trends of both changes were consistent with the RNA-Seq data. Collectively, these transcriptional changes suggest that RTP-2 modulates stress-response and autophagy associated gene programs at the transcript level under the tested condition. The expression of the core autophagy regulator Dram1 was significantly upregulated in the RTP-2 treatment group, which is consistent with a potential involvement of autophagy-related pathways; however, functional activation of autophagy or mitophagy cannot be concluded without direct assays [36]. The upregulation of Bmf1 implies that RTP-2 may activate apoptotic or immunomodulatory pathways associated with cellular stress, thereby further contributing to the remodeling of the immune response [37]. In contrast, Bnip3 expression was slightly downregulated in the polysaccharide-treated group (*p* < 0.05), possibly reflecting the suppression of negative regulatory pathways under this stimulation, which may help maintain immune activation. The expression of Bnip3l, another gene involved in mitochondrial autophagy, showed no significant change (*p* > 0.05), indicating that it is not a major responsive gene to RTP-2 treatment. This further highlights the specificity and selectivity of the regulatory effects observed in RTP-2.

In conclusion, RTP-2 significantly regulated the expression of key autophagy and immune response genes, such as Dram1 and Bmf1, revealing a clear direction for the molecular regulation of the effective enhancement of immune system function. Notably, the expression of the mitochondrial autophagy-related gene Bnip3 was significantly downregulated, whereas the expression of Bnip3l remained unchanged, reflecting a differential targeting mode of regulation. These results highlight the precision and selectivity of the RTP-2 immunoregulatory mechanism. Collectively, these findings provide a solid molecular basis for the potential application of RTP-2 as an immunomodulator.

## 4. Conclusions

This study investigated the immunostimulatory effects of two structurally different polysaccharides from *Rhodomyrtus tomentosa* on RAW264.7 macrophages. The results indicated that RTP-2 exerted significant immunostimulatory effects on RAW264.7 macrophages, manifested as enhanced phagocytic capacity, increased nitric oxide and reactive oxygen species production, and elevated secretion of cytokines (TNF-α, IL-6, IL-1β). Analysis of RNA-seq data using WGCNA revealed activation of the MEturquoise co-expression module comprising 222 hub genes in RTP-2–treated RAW264.7 macrophages. Functional enrichment analysis indicated that this module primarily participates in the mitochondrial autophagy–immune regulation network, with key genes including Dram1, Bmf1, Bnip3, and Bnip3l. Simultaneously, it synergistically remodels the Hspa1b-centered protein homeostasis/stress network and the Sarm1-associated innate immune signaling network associated with Sarm1. Quantitative real-time polymerase chain reaction (RT-qPCR) results indicated that Dram1 expression increased approximately 125-fold, Bmf1 increased approximately 18-fold, Bnip3 was significantly downregulated, and Bnip3l showed no significant change compared to untreated controls. Collectively, these findings indicate that RTP-2 selectively and controllably enhances macrophage immune function, providing a molecular basis for its further development as a macrophage-targeted immune enhancer. This pattern supports a plausible link between RTP-2 exposure and stress/autophagy-associated transcriptional programs in macrophages under the tested condition. A *Peganum harmala* polysaccharide has also been reported to exert similar effects by modulating autophagy-related responses in RAW264.7 macrophages [38]. The MECyan module of RTP-1 predominantly comprises ribosome/translation-related genes and shows no enrichment in immune pathways, indicating relatively limited network integration and immune-directedness. Notably, the transcriptomic profiling was performed at a single dose (40 μg/mL) and a single time point (24 h); therefore, the enrichment results represent a condition-specific snapshot and may not capture early/transient or dose-dependent responses. Future studies may further elucidate its potential mechanisms or other biological activities beyond immunity through multi-omics approaches (single-cell transcriptomics, ATAC-seq, metabolomics, and metabolic flux analysis) and receptor-dependent and endocytic routing experiments.

## Figures and Tables

**Table 1:** Primer sequence information.

Primer	Sequence (5-3′)	Product (bp)
Dram1-F Dram1-R	TCATCTCCTACGTGGTCGC CTGCGCCAAGAAATGCAGAG	135 bp
Bnip3l-F Bnip3l-R	CTGGAGCACGTTCCTTCCTC ACAGTGCGAACTGCCTCTTG	111 bp
Bmf-F Bmf-R	CAGAGACTCTTTTACGGCAACG ACTGGTCTGCAATACACTGAAG	157 bp
Bnip3-F Bnip3-R	CTGGGTAGAACTGCACTTCAG GGAGCTACTTCGTCCAGATTCAT	124 bp
mGAPDH-F mGAPDH-R	AGGTCGGTGTGAACGGATTTG TGTAGACCATGTAGTTGAGGTCA	123 bp

Note: F: Forward; R: Reverse.

**Table 2:** Concentration and integrity of RNA in test samples.

Sample	Concentration (ng/μL)	Volume (μL)	Total Amount (μg)	Integrity Value	Testing Conclusion
A1	509	45	22.905	9.8	A
A2	514	45	23.130	9.7	A
A3	243	45	10.935	8.8	A
B1	365	45	16.425	9.8	A
B2	168	45	7.560	9.7	A
B3	293	45	13.185	9.7	A
C1	273	45	12.285	9.7	A
C2	217	45	9.765	9.6	A
C3	256	45	11.52	9.8	A

Note: Control: A1, A2, A3; RTP-1: B1, B2, B3; RTP-2: C1, C2, C3. A: The quality of the samples satisfied the quality requirements for library sequencing, and the total amount satisfied the need for one or more library constructions. B: Sample quality does not fully satisfy the library building sequencing requirements and can try to build a library. C: Sample quality does not satisfy the library building sequencing requirements and is therefore not recommended for use.

## Data Availability

The original contributions presented in this study are included in the article. Further inquiries can be directed to the corresponding authors.
